# Analysis of the rate of working women’s acceptance of partner violence and cultural reflections in Cypriot society

**DOI:** 10.3389/fpsyg.2022.962423

**Published:** 2023-01-04

**Authors:** Neşe Başak, Nergüz Bulut Serin

**Affiliations:** Faculty of Education, European University of Lefke, Lefke, Turkey

**Keywords:** violence, women, acceptance of violence between couples, culture, society

## Abstract

This case study aims to measure the level of acceptance of violence among women and couples and to reveal the level of acceptance within and outside the families of women who have participated in working life. The concepts of anger and violence, physical, sexual, psychological, emotional, and economic violence, which are the sub-components of violence, violence in dating, and marital relations are examined separately according to Feminism and Social Learning Theory approaches. The Working Group was formed by applying questionnaires to 50 participants from the cities of Kyrenia, Güzelyurt, Lefke, Nicosia, and Magusa and determining the 20 participants with the highest level of acceptance of violence. The 11 people who agreed to the study were interviewed for a Dec of 1 week and 5 sessions ranging from 20 to 30 min. It is observed that women who are subjected to violence have a deep sense of helplessness and accept their helplessness, as well as those women resort to various passive solutions, such as silence and avoiding attitudes that make their partner angrier. The sense of helplessness, as a result of chronic depression, makes it inevitable to experience suicide cases. This study will be particularly important in terms of revealing the levels of domestic and non-family violence exposure and acceptance of violence by women who have participated in working life.

## Introduction

According to Aristotle, people are social animals that must cohabit. People coexist to build societies, and each geography has its own set of laws, as well as a shared way of life (Coştu, 2009).

Individuals play certain roles in society, for this reason, the position of the individual and the concept of the individual are an important issue in the provision of social order in the sociology literature (Fichter, 1996, p. 30).

While roles are supplied to individuals by society in the constructivist-functionalist approach, roles are not imposed on individuals in the social-psychological approach. On the contrary, role is a behavior determined by the individual, which changes according to the individual’s perception of his environment. These roles are learned through observation and imitation in the socialization process (Fichter, 1996, p. 32–33).

Unlike other social status, gender is an inherited position, and every community has its own set of values and gender interpretations. The study of masculinity, in particular, is predicated on the theory that there is a difference between an individual’s sex and gender (Pascoe, 2015). Roles given to women by society separated them from social life and limit their living space. In addition to women’s participation in public life, from politics to the workplace, they also encounter a serious problem, violence.

Violence is one of the most pressing issues of our time, affecting millions of individuals physically and mentally and resulting in thousands of fatalities, injuries, and psychoanalysis each year. Women’s violence is a major public health issue and a violation of their fundamental rights. The social-cultural settings, foregrounds, and social interactions of people all have a role in the emergence of violence, both explicitly and implicitly. The offender, victim, and action are the three dimensions of violence. One out of every three women around the globe is subjected to some form of violence, the most common of which is psychological assault. On the other hand, it has been noted that most women do not talk about the violence they have experienced and that only a small percentage of women seek medical or legal assistance. Some reports back up the theory that violence is generally internalized and tolerated by women (WHO, 2014).

In the study, in order to determine the level of acceptance of gender roles and violence against women, Gender Roles Attitude Scale, Rosenberg Self-Esteem Scale, Attitude Scales for Violence in Dating, Aggression Scale, Couples Violence Acceptance Scale, and Belief Inventory in Relationships were applied to the participants. Among the scales applied to the participants in important centers of the TRNC such as Girne, Nicosia, Lefke, Magusa, and Güzelyurt, those with the highest level of acceptance of violence were determined. A 5-session interview was held with those who accepted the interview among the participants with a high level of acceptance of violence.

Many studies have been found in the world and in Turkey about women who have problems in communication within the family and between spouses. However, although there are issues such as inequality between men and women, gender roles, and violence against women, there are no studies on women in the TRNC. There is a need for further studies in this area in the TRNC. This need is an important need that will benefit society. Working in this field will provide social contribution and benefit to both the women of the country and the country. In this way, more qualified studies will be carried out on these issues, in which women are victims, and measures will be taken or special rights will be provided to women. In this study, it is thought that all of the women participating in the research will benefit from an increase in their awareness levels, hopes, awareness levels, and self-confidence and reach the information they need in this field.

## Literature review

The problem of gender is addressed in depth in this section of the study, and the inequity of gender roles is discussed. The role of women in work life, aggression between couples, and violence against women are then investigated.

### Gender problem

Gender and sex are two entirely distinct concepts. While sex is a notion that describes the physiological status of women or men and is used to symbolize their biological traits, gender is a concept that varies by civilization and stresses the roles and expectations of society on genders.

Gender inequality is a problem that arises from the historical process and affects people all over the world. The values, duties, and patterns assigned to men and women are hierarchically separated, in keeping with the assumption that men are “superior and pioneer.” In this regard, as men determine the place of women in society, males also determine women’s participation.

### Constructing gender roles

The socialization process occurs in a society based on values and social norms. From infancy until adulthood, this is a learning process that is conveyed to individuals with various factors. In this process, role models are quite crucial. Gaining gender roles begins with increasing awareness of children. Once the children become aware, their social environment waits to train them gender-related facts. For example, the mother’s constant presence in the kitchen and the father’s frequent visits to public places, and the dominant colors in the home all have a role in the child’s gender perception. As a result, people unconsciously express their gender at the point of association. The family plays a significant role in the development of manly and womanly perceptions throughout this time period.

### Gender roles in Turkey

The construction of gender roles in Turkish society can be examined in three periods: pre-Islam, post-Islam, and Republican periods, respectively. It has been noted that in pre-Islamic Turkish society, men and women had equal rights and women had an important role in the political arena and in the family. According to Zia Galkal, “No tribe among the ancient tribes respected for the women gender as much as Turkish society did” (Bingöl, 2014, p. 111).

Following Islam, men and women’s gender roles became more clearly defined. In this process, women were separated from society and accepted as substitutes for men. In general, while Islam provides women a powerful role and status, misinterpretation of numerous verses and narrations has led to women being treated as a commodity in some circumstances. In the Republican era, men and women were treated equally. Women were given the right to vote and equal rights as a result of the reforms implemented in this context. The most valuable female role, on the other hand, was still deemed the mother role. Nonetheless, each year, women’s educational achievement and political involvement have risen dramatically.

### Male and female inequality in Turkey

Turkey ranks 109th out of 118 nations in terms of gender-based income disparity, according to international statistics, and gender inequality is prevalent in education and business. The fact that some girls are married at a young age is a significant barrier to obtaining the requisite education and entering the workforce. According to statistics gathered from numerous research, two out of every three people in Turkey acknowledge that men and women are treated unequally.

### Violence and types of violence against women

While the twentieth century is commonly referred to as the information era, social scientists refer to it as the century of violence. In feminist social networks and discourse, “violence” is now defined as violence against women. The social attitude toward women, as well as the context in which this attitude exists, demonstrate that violence against women is both normal and acceptable.

Sexual violence, according to the United Nations, is any act of gender-based violence that causes physical, sexual, or psychological harm to women, including threats, arbitrary deprivation of liberty, or coercion in both private and public life. Women’s violence appears to be a gender-based problem. According to the relevant literature review, violence against women is generally classified as physical violence, sexual violence, psychological violence, and economic violence.

• Physical violence

Since it is more measurable than other forms of violence, physical violence is used to define and communicate violence against women. Individuals who are subjected to physical violence can be more easily identified and accurately assessed. In other words, physical violence stands out more than other forms of violence. Physical violence against women includes swipe, kicking, pulling, injuring, or preventing required treatment (Afşar, 2015).

• Sexual violence

This type has been a subcategory of physical violence for many years, but it has been separated from physical violence due to its emergence and effects. Sexual violence can be generally explained as forced sexual intercourse. The most harm-causing form of this type is rape, which can be found both in singles, in dating relationships and in married couples. Sexual violence includes forced sexual intercourse with other people, forced marriage, forced childbirth, forcing abortions, damaging their genitals, verbally or visually sexual harassment, mocking their gender characteristics, etc. Several studies argue that honor killings should also be evaluated as part of sexual violence (Afşar, 2015).

• Psychological violence

This type, also known as emotional violence, is defined as systematic psychological pressure, exploitation, insulting, trying to control emotions, and isolating the individual from society. Psychological violence includes threatening women, not allowing them to make their own decisions, comparing, threatening, teasing, swearing, intimidating, preventing them from going out and meeting with friends, belittling their attitudes and behaviors, etc. (Afşar, 2015).

This type of violence may not have visible effects like other types of violence. However, because of the disregard for women and mental breakdown, it has a greater impact than physical violence. This sort of violence is more prevalent in married couples.

• Economic violence

The most emerging violence is economic violence which is defined as forced work of women to earn income or preventing them from working. Economic violence includes not giving wife money, preventing her from using the money, taking her money or bank card back, not letting her work, confiscating her properties, not acting based on the woman’s opinion in economic affairs and expenses within it, etc. (Afşar, 2015).

## Method

The method section includes the design, population, and sampling of the research, data collection tools and techniques used in the research, development of the measurement tool, data collection process, and data analysis. Besides, information about statistical analysis techniques will be given.

### The research model

Quantitative research, which is defined as the systematic investigation of events by using sampling methods, collecting quantitative data, and applying statistical, mathematical, or computational techniques, is used in the study. The following scales are used:

• Gender Roles Attitude Scale
• Rosenberg Self-Esteem Scale
• Attitude Scales for Violence in Dating
• Aggression Scale
• Couples Violence Acceptance Scale
• Belief inventory in relationships

Then, interviews were held with those who accepted, and in-depth information was obtained.

### Population and sampling

The study group was formed by applying questionnaires to 50 participants from the cities of Kyrenia, Guzelyurt, Lefke, Nicosia, and Magusa and determining the 20 participants with the highest level of acceptance of violence. Kyrenia, Güzelyurt, Lefke, Nicosia, and Magusa in studies with 10 people from each city and the acceptance of violence as a result of the evaluation of the questionnaire with the highest level of the 20 participants designated to perform these people were contacted about the violence they see a conversation session. With 11 participants who agreed to participate in the research, five sessions were held at an interval of 1 week.

### Data collection tool

To begin with, a questionnaire is prepared utilizing the previously indicated scales. In addition, a section describing the participants’ demographic data has been included. The questionnaire will be applied to Kyrenia, Lefkoşa, Magusa, Lefke, and Güzelyurt.

### Collection of data

The data collection section has been in the form of a survey study conducted in Turkish Republic of Northern Cyprus in Kyrenia, Lefkoşa, Magusa, Lefke, and Güzelyurt. Before the application of the questionnaire, an oral instruction was given to the 10 people from each region to participate in the survey about the importance of the research and the effect of their sincerity in answering the results of the research and the process of answering.

### Data analysis

SPSS 23.0 package program will be used to investigate whether there are significant differences in the perception of the sub-dimensions of the scale based on the validity and reliability analysis of the scale and the demographic data of the employees.

## Findings

This research was carried out by a face-to-face survey among 50 volunteer female participants in 2019. The women who participated in our research were selected in groups of 20% (*n* = 10) from a random sample of women from Kyrenia, Guzelyurt, Lefke, Nicosia, and Magusa. The average age of the participants was 28.82 ± 6.18 (minimum 19, max 44).

The mean scores according to the answers given to the questionnaire were on the Couples Violence Acceptance Scale of 14.02 ± 3.03, the Gender Roles Scale of 94.92 ± 8.40 and the Rosenberg Self-Esteem Scale of 20.52 ± 5.32. In the Attitudes Toward Violence Against Flirting scale, the averages of the participants are as follows; Attitudes Toward Psychological Violence in Dating 32.68 ± 4.95, Physical Flirtation Applied by Men in Flirtation Severity Attitudes Scale 18.10 ± 4.82, Women’s Attitude Scale for Psychological Violence in Dating, Women’s Attitudes to Physical Flirting Severity Scale 23.60.

In addition, the participants’ Faith Scale averages are as follows; 42.14 ± 4.37, Desperation 26.58 ± 3.54, and Lovelessness 15.26 ± 2.36. Furthermore, the average scores of the sub-dimensions of attitude toward Violence Against Dating of the women who participated in our research are as follows; Social Value 72.22 ± 13.99, Career Values 31.46 ± 3.59, Intellectual Values 43.66 ± 3.60 Spirituality 24.26 ± 3.26, Materialist Values 16.98 ± 3.88, Human Dignity 23.16 ± 1.71, Romantic Values 21.04 ± 2.68, Freedom 20.92 ± 2.38, Price rating 11.02 ± 2.84.

According to the analysis results regarding the relationship between demographic characteristics and the scales used, statistically significant differences were found in the sub-factor of freedom of the marital status and values scale (*p* < 0.0001). Single and widowed participants were found to have higher freedom sub-factor scores than married participants (Table 1). According to the *post hoc* Tamhane test, it was determined that single scores are statistically significant (*p* < 0.001) and higher than married people.

**TABLE 1:** Relationship between marital status, having children, and education level with related scales.

Scales	Marital status	*n*	Average	*x* ^2^	*p*
Values scale (freedom scale)	Single	20	22.45 ± 2.13	–16.156	<0.0001
	Married	25	19.64 ± 1.93		
	Widow	5	21.2 ± 1.52		

	**State of having children**	***n***	**Average**	***z***	***p***

Values scale (freedom)	No kids	25	22.00 ± 2.16	–3.933	<0.0001
	Kids	25	19.64 ± 1.77		
Values scale (romantic values)	No kids	25	21.64 ± 2.89	–2.083	0.037
	Kids	25	20.44 ± 2.36		

	**Education level**	***n***	**Average**	***x* ^2^**	***p***

Values scale (career value)	High school	12	29.66 ± 4.18	–3.933	<0.0001
	University	27	31.07 ± 2.92		
	Postgraduate	11	34.36 ± 2.87		
The scale of a woman’s attitude toward physical violence in dating	High school	12	24.08 ± 2.23	–2.083	0.037
	University	27	23.74 ± 3.47		
	Postgraduate	11	21.18 ± 3.31		

A statistically significant difference was found between having a child and the freedom and romantic values sub-dimensions of the values scale (*p* < 0.0001, *p* = 0.037). Freedom and romantic value scores were statistically significant and higher than the participants with and without children (Table 1).

A statistically significant difference was found between the education level and the career sub-dimension of the values scale and the women’s attitude toward physical violence while dating (*p* = 0.0007, *p* = 0.044). As the education level increased, the career sub-dimension of the values scale increased, while the women’s attitude scale score toward physical violence decreased (Table 1). According to the *post hoc* Tamhane test, those with postgraduate education were statistically significant (*p* = 0.015) and higher than those with a high school education or below (Table 1).

A statistically significant difference was found between the working conditions of the participants and the acceptance scale between couples, the scale of attitude toward physical violence by women while dating, the helplessness of belief in the relationship and the helplessness scale in all sub-dimensions, and the Rosenberg self-esteem scale (*p* = 0.001, *p* = 0.009, *p* = 0.005, *p* = 0.005, *p* = 0.001). The scores of the non-working participants compared to the working participants were statistically significant and higher on all of the mentioned scales (Table 2).

**TABLE 2:** Relationship between employment status and scales.

Scales	Employment status	*n*	Average	*x* ^2^	*p*
The scale of admission of inter-couple violence	Unemployed	14	16.28 ± 2.99	–3.379	0.001
	Employed	36	13.13 ± 2.58		
The scale of a woman’s attitude toward physical violence in dating	Unemployed	14	25.35 ± 3.10	–2.605	0.009
	Employed	36	22.44 ± 3.06		
Faith in the relationship (desperation)	Unemployed	14	29.00 ± 2.93	–2.809	0.005
	Employed	36	26.05 ± 3.45		
Faith in the relationship (total)	Unemployed	14	44.64 ± 3.83	–2.832	0.005
	Employed	36	41.16 ± 4.22		
Rosenberg self-esteem scale	Unemployed		24.64 ± 4.21	–3.448	0.001
	Employed	36	18.91 ± 4.86		

There is a statistically significant difference between the age of the participants and gender roles, intellectual values, values scale, spirituality, freedom, and the sub-dimensions of romantic values (*p* = 0.009, *p* = 0.017, *p* = 0.017, *p* = 0.027, *p* = 0.037). As the age of the participants increased, the scores of the gender role scale score and the moral sub-dimension scores of the values scale were statistically significant and high, while the intellectual values, freedom, and romantic values sub-factors of the values scale scores were statistically significant and low (Table 3).

**TABLE 3:** Relationship between age and scales.

Scales	Age	*n*	Average	*Z*	*p*
Gender roles scale	19–29	32	92.46 ± 5.62	–2.602	0.009
	30–44	18	99.27 ± 10.69		
Values scale (intellectual values)	19–29	32	44.40 ± 3.37	–2.396	0.017
	30–44	18	42.33 ± 3.60		
Values scale (spirituality)	19–29	32	23.56 ± 2.73	–2.392	0.017
	30–44	18	25.55 ± 3.82		
Values scale (freedom)	19–29	32	21.46 ± 2.40	–2.211	0.027
	30–44	18	19.94 ± 1.92		
Values scale (romantic values)	19–29	25	21.65 ± 2.75	–2.598	0.037
	30–44	25	19.94 ± 2.23		

Statistically significant differences were found in the participants’ relationship duration and gender roles scale, and in the intellectual values, spirituality, and romantic values sub-factors of the values scale (*p* = 0.036, *p* = 0.011, *p* = 0.003, *p* = 0.021). The gender roles scale and morality sub-factor scores in the values scale of the participants with a relationship period of 5 years or more were statistically significant and higher than the participants with a relationship period of 1–4 years. On the other hand, in the values scale, the sub-factor scores of intellectual values and romantic values were found to be statistically significant and low (Table 4).

**TABLE 4:** Relationship between relationship duration and scales.

Scales	Relationship time	*n*	Average	*z*	*p*
Gender roles scale	1–4 between years	29	92.58 ± 5.01	2.098	0.036
	5 years and above	21	98.14 ± 10.9		
Values scale (intellectual values)	1–4 between years	29	44.75 ± 3.15	2.544	0.011
	5 years and above	21	42.33 ± 3.69		
Values scale (freedom)	1–4 between years	29	23.27 ± 2.75	2.972	0.003
	5 years and above	21	25.61 ± 3.49		
Values scale (romantic values)	1–4 between years	29	21.65 ± 2.84	2.309	0.021
	5 years and above	21	20.19 ± 2.24		

## Results and suggestions

The study entitled “Examining the Degree of Acceptance of the Level of Violence against Working Women” was conducted on 10 women from Kyrenia, Guzelyurt, Lefke, Nicosia, and Magusa in Cyprus. Different studies have investigated inter-couple violence (Swart et al., 2002). Violence perceptions differ based on gender, age, etc. Based on the TURKSTAT data (2012, 2016), most women in Turkey become victims of domestic violence. Different factors, such as excessive alcohol drinking (Watkins et al., 2014), substance use (Merghati-Khoei et al., 2014), race (Powers et al., 2012), unsafe attachment (Craparo et al., 2014), and exposure to violence in early childhood (Yount et al., 2014) are effective in causing disorder. The levels of anxiety and depression reduce emotional wellbeing and self-esteem (Callahan et al., 2003) due to the acceptance of violence among couples, which creates thoughts about eating disorders, substance use, and suicide (Ackard and Neumark-Sztainer, 2002; Ackard et al., 2007). Women who don’t have confidence and self-esteem tolerate violence instead of solving violence (Kutluk, 1994). This study showed statistically significant differences between the marital status of the participants and lower freedom. The participants who were single and widowed showed higher freedom. The freedom and romantic scores were statistically significantly elevated compared to the participants with children. The higher learning status increased the career value and decreased the attitude toward physical violence among women. There was a statistically significant relationship between the education of the participants and the acceptance of violence. The results showed that the higher the age of the participants, the higher the gender role scale score and the morality subfactor scores. There were low scores for intellectual values, freedom, and romantic values sub-factors. The smaller the gender roles scale, the higher the morality subfactor. The intellectual values and romantic values were significantly lower among the participants whose relationship was 5 years or above. Some participants say that they were exposed to violence due to the addiction of their husband to gambling and being in a car accident and disruption. Only one participant said that she wanted to end the relationship. Most of the participants stated that they experienced physical violence (63%), and three (27%) stated that they were subjected to psychological violence. 18% of the participants also say they are exposed to economic and psychological violence.

Regarding the causes of violence, the answers include women’s health problems, economic, cultural, and social differences between spouses, a stressful environment, family education, patriarchal structure, belief in the violent representation of love, drug addiction, gambling addiction, and alcohol addiction. Regarding the effect of their environment and the guidance of themselves or their partners, there were often suggestions to comfort those who mentioned the subject, but there was no concrete intervention. Three (27%) stated that they were subjected to violence and could not share it with anyone. One (9%) stated that her husband and her own family were financially supportive. Regarding the kinds of problems, the answers were health problems, economic problems, cultural and social differences, the spouse’s love of violence, and the spouse’s stressful and busy work environment. 45% expressed the difficulty in coping with physical violence. One participant expressed difficulty in dealing with psychological violence while coping with economic violence. All participants (91%) expressed that they were psychologically worn out. Only one participant said that she was physically worn out. The answers “love” and “respect” were measured. Socio-economic status was considered by the participants. All participants but one believes that nothing deserves violence. Four participants said they understood that they accepted violence. The main reason for acceptance was that they didn’t want to lose. Four people answered “desperation,” four people answered “excessive love,” and three people answered “economic dependence.” “Economic violence” almost affected their freedom, and participants thought that their freedom was restricted. Four people said that they did not want to lose and got tired, and three people did not want to disrupt their families. One person mentioned “fear of humiliation” as the main reason for acceptance. Ten participants (91%) said they never considered violence as a sign of pleasure, a sign of love, while 1 participant said: “But I’m starting to like it now.” All participants but one stated that they could not solve the problems of violence and even caused other problems. Nearly half of the participants (45%) did not ask anyone for help when they were exposed to violence. Other participants (55%) said they told their families, neighbors, friends, spouse’s family, and their wife’s family. 82% said that they had lost their confidence. There was no development of violent behavior in the participants (73%). The two participants stated that they had developed a predisposition to violence. One of the participants said that she began to raise her voice against her children, while the other began to restrict her husband. 45% said that they had become silent and insensitive to violence.

Women begin to see violence in the early stages of their marriage or relationship. The study found that participants made some unsuccessful attempts to solve problems. Nearly half of the participants did not ask anyone for help when they were exposed to violence and did not tell others about the violence. Fifty-five percent of those who told their families and others about the violence could not solve the problem. The participants suffered a severe loss of confidence after being exposed to violence. Similarly, in the first study on violence against women in the TRNC conducted by Çakıcı et al. (2001) in the TRNC, violence against women is intense, but these issues are not discussed in public because they are considered private. It is seen that the violence applied is physical and verbal violence, and divorces due to violence are low due to women’s lack of economic independence and lack of family support. Likewise, in the study of violence against women in the TRNC conducted by Düşünmez (2004), it is seen that women are exposed to physical and psychological abuse by their husbands and families, but they prefer to hide the violence they have suffered.

According to Uludağ (1998), issues such as women’s lack of self-confidence, their economic dependence, and the absence of support mechanisms against violence show that there is no gender equality in Northern Cyprus, as in the rest of the world. This situation can also be seen in the language of the media in the written press in the TRNC. Aliefendioğlu (2009) states that the TRNC print media is a platform where men address men with a sharp masculine language. In the TRNC, where a patriarchal social structure is observed, the fact that the media also determines men as the main subject, that all articles are published far from an egalitarian language and that their attitude is negative in conveying news to the mass in cases of violence against women is an indicator of this (Ceylanli and Kanli, 2020).

Violence against women, which is basically based on gender inequality, is an issue that needs to be addressed and given importance all over the world. In fact, according to the 2020 data of the World Health Organization (WHO), it is seen that one out of every three women in the world has been exposed to physical, verbal, or sexual violence from her husband or family (WHO, 2020). It is also recommended that patriarchal values and sexist slurs as an expression of manhood should be avoided (Peter and Raj, 2021).

Especially in societies where patriarchal culture is more dominant, such as the TRNC, women are more likely to be subjected to violence by men. According to the feminist theory, this situation arises from the power and resource imbalance in the relations between men and women, approval of violence, alcohol and substance use, and childhood traumas (Yodanis, 2004). However, the prevention of violence against women, which poses a high risk for public health and human rights, and the development of public health interventions attract more attention because violence is ignored in patriarchal societies (Manchikanti Gomez, 2011).

Social support programs can be prepared to improve the social relationships of women exposed to violence in studies. Women who are subjected to violence and accept violence should seek professional help to avoid violence. Identifying the factors that lead individuals to accept violence will contribute to subsequent studies. Further studies should be conducted to help individuals learn to communicate appropriately from a very early age. The misconception of problem-solving should be emphasized by paying attention to parent education and understanding gender roles and violence.

## Data availability statement

The raw data supporting the conclusions of this article will be made available by the authors, without undue reservation.

## Ethics statement

The studies involving human participants were reviewed and approved by the Ethical Committee Board of the European University of Lefke. The patients/participants provided their written informed consent to participate in this study.

## Author contributions

Both authors listed have made a substantial, direct, and intellectual contribution to the work, and approved it for publication.
